# How Biotin Induces Misleading Results in Thyroid Bioassays: Case Series

**DOI:** 10.7759/cureus.4727

**Published:** 2019-05-23

**Authors:** Samih A Odhaib, Abbas A Mansour, Nazar S Haddad

**Affiliations:** 1 Diabetes and Adult Endocrinology, Faiha Specialized Diabetes, Endocrine and Metabolism Center, College of Medicine, University of Basrah, Basrah, IRQ; 2 Diabetes and Endocrinology, College of Medicine, University of Basrah, Basrah, IRQ; 3 Iraqi Board of Medical Specializations In Clinical Chemistry (fibms. Chem. Path.), American Association for Clinical Chemistry (faacc), College of Medicine, University of Basrah, Basrah, IRQ

**Keywords:** biotin, thyroid bioassay, immunoassay, grave's disease, pseudohyperthyroidism

## Abstract

Biotin is widely available over the counter in different doses and is used medically in the management of hair and nail problems. Recent literature suggested the use of high doses of biotin for the treatment of progressive multiple sclerosis. We present four cases that show a misleading increase in thyroid function tests toward a false diagnosis of Grave's disease after the administration of 20-30 mg biotin for different periods. All the four cases are free of the signs and symptoms of hyperthyroidism, and all of their results returned to baseline values within 24-48 hours after biotin withdrawal. The assemblage of these cases, thyroid assay results, and biotin doses had occurred by chance, with no selection bias, and they represented all cases of biotin interference with thyroid assays in our center during this year. The first case is a 23-year-old female who was diagnosed with Grave's disease during a routine checkup after she ingested a prescribed 20 mg biotin per day for three months for excessive hair fall. The second case is a 19-year-old female with hair and nail problems associated with iron deficiency anemia. She administered a self-prescribed biotin dose of 20 mg a day for a month. She asked an endocrinologist's opinion about a recent increase in her thyroid function tests, with no signs of hyperthyroidism. The third case is a 45-year-old man with near-total thyroidectomy for retrosternal multinodular goiter with compressive symptoms. His usual levothyroxine dose had been decreased from 100 to 50 microgram per day, after which he felt unwell and gained four kilograms, with signs and symptoms of hypothyroidism. His investigations were consistent with hyperthyroidism while his signs were of hypothyroidism, which was illogical. He was administered 30 mg medically prescribed biotin for nail changes due to recently diagnosed psoriasis. The fourth case is that of one of the authors who volunteered to take 30 mg of biotin daily for one week. His initial investigations were in the normal range but changed within this period to be Grave's disease-like, with no signs or symptoms. In conclusion, the ingestion of 20 mg or more of biotin may lead to a clinically relevant thyroid assay interference. The clinicians must take this point in consideration before assessing the results of any thyroid function tests.

## Introduction

Biotin is a negatively charged, water-soluble B complex vitamin (B7, B8, or H) [1] and an essential cofactor in the activation of many biotin-dependent carboxylases [2]. The primary site of biotin absorption is the intestinal brush border, with a 110 minutes plasma half-life [1]. Biotin is covalently bound to proteins, polypeptides, and low molecular weight antigens, forming the biotinyl-lysine complex after the digestive breakdown of biotin-containing protein [1,3].

Generic formulations of biotin, up to 10 mg (100-200 times higher than the standard dietary reference intake (DRI) estimates of 17-70 µg), are widely available over the counter [2]. Recently, the high-dose biotin (HDB) (100 mg to 300 mg/day, which is 10,000 times the DRI) was vital in the management of progressive multiple sclerosis [4]. Biotin affects the acetyl CoA carboxylase activation mechanism as a cofactor in myelin production through the promotion of oligodendrocytes-dependent remyelination by enhanced fatty acid synthesis and the prevention of demyelinated axons degradation by increased energy production [5].

There are several, rare, inherited metabolic diseases that can benefit from the HDB (e.g., biotinidase deficiency: 5-10 mg/day; holocarboxylase synthetase deficiency: 30-40 mg/day; biotin-thiamine-responsive basal ganglia disease: 100-300 mg/day) [6-7]. Doses up to 30 mg/day are now widely used for improving hair, nail, or skin condition [6] and for supportive treatment in mitochondrial energy metabolism disorders, lipid disorders, and diabetic peripheral neuropathy [3,7].

There are many new generation thyroid bioassays that depend on the streptavidin-biotin complex as the immobilizing method or affinity tag to improve the sensitivity amplification especially in detecting low analyte levels [8].

We present four cases that illustrate the temporarily marked effect of biotin administration interference with the thyroid bioassays.

## Case presentation

Case 1: mistaken as Grave's disease

DS is a 23-year-old married female who came for the evaluation of accidentally discovered high thyroid function readings during the treatment course for excessive hair fall. She was referred from a fellow dermatologist, to have a second opinion about the possibility of Grave's disease.

On initial interview, DS appeared calm and not in any distress. Her past and recent medical history were noncontributory, apart from the excessive hair fall in the past three months, for which she sought the dermatologist's advice for treatment by topical creams and lotions, alongside with biotin as a hair tonic in a dose of 10 mg twice daily. General and systemic examinations were noncontributory.

Her investigations gave a picture of Grave's disease, yet she was free of symptoms or signs. So, we advised her to repeat the assays (Table 1). Her thyroid ultrasound examination was normal. We reviewed the results and advised DS to quit biotin for the following 24 hours and to repeat the tests afterward to ensure the diagnosis of thyroid bioassay interference by biotin ingestion (Table 1). It took 48 hours for the biotin interference to disappear.

**Table 1:** Thyroid function test of a 23-year-old female with a wrong diagnosis of Grave's disease after 20 mg/day biotin therapy for excessive hair fall.

Investigations	On biotin		Off biotin		Normal values
	October 10^th^, 2018^¶^	October 12^th^, 2018^¶¶^	24 hours after discontinuation^¶¶^	48 hours after discontinuation^¶¶^	
TSH µIU/ml	0.05	0.06	0.52	1.44	0.27-4.2
FT4 ng/dl	2.93	3.11	1.99	1.08	0.93-1.7
FT3 pg/ml	6.09	6.93	4.51	2.31	1.21-4.18
TT4 µg/dl	15.12	14.87	6.44		5.1-14.1
Anti-TPO IU/ml	6.33				0-34
TRAb mIU/ml	4.74	5.0	1.96		<2
Tg ng/ml	19.76				1.40-78
Anti-Tg IU/ml	33.62				0-115
^¶^ Snibe Maglumi 800 immunoassay. ^¶¶^ Roche Cobas e411 immunoassay. TSH: Thyroid-Stimulating Hormone, FT4: Free Thyroxine, FT3: Free Triiodothyronine, TT4: Total Thyroxine, Anti-TPO: Antithyroid Peroxidase, TRAb: Thyrotropin Receptor Antibody, Tg: Thyroglobulin, Anti-Tg: Anti-Thyroglobulin.					

Case 2: pseudohyperthyroidism

ZR is a 19-year-old married female who had a history of iron deficiency anemia (IDA) and polycystic ovary syndrome (PCOS), with bothersome androgenic alopecia, hirsutism, and nail changes. A fellow gynecologist referred ZR for assessing recently discovered elevated thyroid function tests.

On initial interview (November 24, 2018), her assessment was noncontributory, apart from her complaint of hair, nail, and infertility problems. In the previous three months, she took regular 300 mg ferrous sulfate daily for the treatment of her IDA, with a treatment regimen for her PCOS in the form of spironolactone tablet 100 mg twice daily, finasteride tablet 5 mg once daily, and metformin tablets 1700 mg daily. She confessed to ingest a 20 mg self-prescribed biotin daily for the last month as a hair tonic, as instructed by many hair care websites. Her initial investigations (three months ago) included an optimal basal thyroid function test and ultrasound, with biochemical and clinical evidence of PCOS and IDA.

After repeating the thyroid function tests in our laboratory, we believed that the main culprit was the 20 mg biotin that caused such interference with the thyroid bioassay (pseudohyperthyroidism). We advised ZR to discontinue biotin and to repeat the test after 24 hours. Some of her tests returned to baseline after 24 hours while the free thyroxine (FT4) needed 72 hours to return to its baseline level, as shown in Table 2.

**Table 2:** A 19-year-old female with pseudohyperthyroidism due to self-prescribed 20 mg/day biotin for hair and nail changes in iron deficiency anemia and polycystic ovary syndrome

Investigations	Initial investigations Aug 30, 2018	On biotin		Off biotin			Normal values
		Nov 22, 2018	Nov 24, 2018	24 hours after	48 hours after	72 hours after	
TSH µIU/ml	0.86	0.04	0.05	1.04	1.11		0.27-4.2
FT4 ng/dl	1.63	3.23	3.48	2.55	2.30	1.04	0.93-1.7
FT3 pg/ml			4.01	2.24			1.21-4.18
TT4 µg/dl	14.00	22.12	20.17	10.30			5.1-14.1
TT3 ng/ml	0.9	4.21	4.56	1.56			0.8-2
Anti-TPO IU/ml			3.22				0-34
TRAb mIU/ml			0.68				<2
^¶^ Roche Cobas e411 immunoassay. TSH: Thyroid-Stimulating Hormone, FT4: Free Thyroxine, FT3: Free Triiodothyronine, TT4: Total Thyroxine, TT3: Total triiodothyronine, Anti-TPO: Antithyroid Peroxidase, TRAb: Thyrotropin Receptor Antibody.							

Case 3: false reduction of the thyroxin dose

HA is a 45-year-old man who underwent a near-total thyroidectomy on April 22, 2018, for retrosternal multinodular goiter with compressive symptoms. He was administered 100 µg levothyroxine daily, with a good clinical outcome. He had regular checkups, as adviced by his surgeon. HA was well until September 16, 2018, when his surgeon decreased the thyroxine dose to 50 µg/day, after high readings of thyroid function tests during his routine visit. HA did not feel well, and he was exhausted all the time, with four kilograms' additional weight gain, constipation, and disturbed sleep with low mood.

The surgeon referred HA for an endocrinologist's opinion on January 2, 2019. His previous investigations revealed hyperthyroidism that ran in discrepancy with the patient's symptoms of hypothyroidism, which was evident clinically during the initial assessment. We performed another set of thyroid tests that gave the diagnosis of TRAb-positive Grave's disease, which was illogical for us and HA.

After again going through the drug history, we discovered that HA was additionally administered a 30 mg biotin tab daily as prescribed treatment for his nail changes due to recently diagnosed psoriasis. HA never mentioned biotin in his drug history because he believed that biotin is a vitamin, not a drug. We advised him for biotin withdrawal and repeated investigation after 24 hours.

After biotin discontinuation, the TSH returned to normal in 24 hours and was on the low side after 48 hours while the FT4 and FT3 needed 48 hours to return to baseline. The TRAb turned negative after 72 hours. HA received his usual 100 µg/day thyroxine again. We scheduled the next visit to be on February 20, 2019, at which there were no signs of the original complaint with the pre-biotin thyroid hormone level (Table 3).

**Table 3:** Wrong diagnosis of Grave's disease in a 45-year-old male with near-total thyroidectomy on 100 µg/day thyroxine due to 30 mg/day biotin therapy for nail changes of psoriasis.

Investigations	Initial investigations^¶^				On biotin^¶¶^	Off biotin^¶¶^			Feb 20, 2019	Normal values
	Before operation April 22, 2018	To start thyroxin 100 µg/d May 20, 2018	July 1^st^, 2018	September, 16,2018 thyroxin 50 µg/d	January 2, 2019	24 hours after	48 hours after	72 hours after and restart thyroxin 100 µg/d		
TSH µIU/ml	1.24	13.13	5.01	0.05	<0.05	2.11	6.34	6.92	3.59	0.27-4.2
FT4 ng/dl	0.96	0.52	1.04	2.67	5.03	3.32	1.42	0.90	1.45	0.93-1.7
FT3 pg/ml	1.52	0.91	3.11	2.09	12.11	5.05	2.15	0.91	3.06	1.21-4.18
TT4 µg/dl	5.55		9.07	15.4	15.31	10.09				5.1-14.1
TT3ng/ml	0.98									0.8-2
Anti-TPO IU/ml	6.17				8.77					0-34
TRAb mIU/ml	0.857				9.3	4.233	3.32	1.68	0.92	<2
Tg ng/ml	52.09				71.30	22.28				1.40-78
Anti-Tg IU/ml	92.96				54.81					0-115
^¶^ Snibe Maglumi 800 immunoassay. ^¶¶^ Roche Cobas e411 immunoassay. TSH: Thyroid-Stimulating Hormone, FT4: Free Thyroxine, FT3: Free Triiodothyronine, TT4: Total Thyroxine, TT3: Total triiodothyronine, Anti-TPO: Antithyroid Peroxidase, TRAb: Thyrotropin Receptor Antibody, Tg: Thyroglobulin, Anti-Tg: Anti-Thyroglobulin										

Case 4

One of the authors volunteered to take 30 mg of biotin daily for one week to simulate - to an extent - the possible biotin dose interference in the three aforementioned cases. The results of his initial investigation done in his annual checkup on February 24, 2019, are shown in Table 4. There was a biochemical picture of hyperthyroidism, with no signs or symptoms to be experienced by him. All the investigations returned to average baseline values after 24 hours of discontinuation of biotin.

**Table 4:** Thyroid function tests of a 41-year-old adult endocrinologist who volunteered to be administered 30 mg/day biotin for a week

Investigations^¶^	Initial investigations Feb 24, 2019	On biotin	Off biotin	Normal values
		Feb 26, 2019	24 hours after	
TSH µIU/ml	1.45	0.8	1.33	0.27-4.2
FT4 ng/dl	1.36	1.94	1.24	0.93-1.7
TT4 µg/dl	10.06	7.32	6.94	5.1-14.1
TT3 ng/ml	1.4	2.77	1.91	0.8-2
Anti-TPO IU/ml	15.47	54.87	12.5	0-34
TRAb mIU/ml	0.770	6.97	1.2	<2
Tg ng/ml	7.12	6.32		1.40-78
Anti-Tg IU/ml	<10.00	25.18	7.45	0-115
^¶^ Roche Cobas e411 immunoassay. TSH: Thyroid-Stimulating Hormone, FT4: Free Thyroxine, TT4: Total Thyroxine, TT3: Total triiodothyronine, Anti-TPO: Antithyroid Peroxidase, TRAb: Thyrotropin Receptor Antibody, Tg: Thyroglobulin, Anti-Tg: Anti-Thyroglobulin				

## Discussion

The interference of biotin with the thyroid bioassays is evident in all four cases, and it disappears after biotin withdrawal. Being chemiluminescent biotinylation-based assays with nearly the same normal reference values, the Roche Cobas e411 and Snibe Maglumi 800 bioassays gave results pointing toward the diagnosis of pseudohyperthyroidism and even Grave's disease, led, in part, to wrong medication changes, as in Case 3, and warranted further unnecessary investigations, as in Cases 1 and 2. This interference works in vitro only, with no real clinical effect, i.e., it is biochemical.

The streptavidin-biotin complex represents one of the most robust non-covalent interactions in nature. This complex is not disturbed by multiple washing steps, and biotinylation typically does not alter the biological activity or immunologic specificity when bound to a test molecule [6,9].

This methodology is currently widely used in several Food and Drug Administration (FDA)-approved immunoassay systems using fully automated platforms, including Access, DxI, and DxC (Beckman Coulter, California, US); the Elecsys, Cobas, and Modular platforms (Roche Diagnostics, Basel, Switzerland); the Isys platform (Immuno Diagnostic System, East Boldon, United Kingdom); the Ortho Vitros platform (Ortho Clinical Diagnostics, New Jersey, US); the Dimension Vista, Exl, Immulite platforms (Siemens Healthineers, Erlangen, Germany), Abbott Architect i2000® (Abbott Diagnostics, Illinois, United States), and Diasorin Liaison XL® (DiaSorin, Saluggia, Italy) due to its small molecular weight of 244.3 Dalton [6,10-11].

Low biotin doses in vitamin supplements are not thought to interfere with bioassays, though some manufacturers mention the interference potential for anyone taking biotin >5 mg/day [11]. This supraphysiological HDB in blood has produced an emerging problem with insidiously misleading immunoassays results (for the biotin-streptavidin binding that accounts for about half of all current immunoassays), fully mimicking and indistinguishable from the typical biochemical picture of Grave's disease and sometimes persisting for several days after biotin withdrawal [3,8,12]. This interference occurs through complex mechanisms that involve either interfering with the analytical system themselves or by influencing their endogenous constituents [9]. The minimal dose required for interference and the degree, duration, and magnitude of this erroneous interference to occur is not known and might be analyte-specific and challenging to assess [9,13].

The artifact result is falsely increased hormone concentrations using competitive assays (e.g., T3, T4, steroid hormones, 25-OH vitamin D) and falsely decreased hormone concentrations using sandwich assays (glycoprotein-regulating hormones) [6,14]. These opposite effects may lead to pseudohyperthyroidism, with apparently elevated free thyroxine and free triiodothyronine and lowered thyroid-stimulating hormone concentration [3,6,12,15-16]. Sharma et al. illustrated some cases with a false elevation of the anti-TSH receptor antibody (TRAb) titers, suggesting Grave's disease, leading to additional inappropriate therapy [14], the effect that was affirmative in two of our patients (Cases 1 and 3).

In the free thyroxine competitive immunoassays, we incubate the serum sample with a ruthenium-linked anti-thyroxine antibody complex, and then the added biotinylated thyroxine will bind unoccupied excess sites on this complex [13,15]. The biotinylated T4-antibody complex then attaches to the streptavidin-coated tube, or microparticles on the solid phase, and generates chemiluminescence signals by the alkaline phosphatase conjugate and chemiluminescent substrate [16-18].

This chemiluminescence is inversely proportional to the free analytes in the sample [12-13,15]. A high biotin concentration in the sample saturates the streptavidin-binding sites and prevents the streptavidin binding to the biotinylated-analyte, resulting in a little or no labeled T4-antibody complex binding to the solid phase and, hence, a falsely elevated free thyroxine level [13,15].

In sandwich immunoassays, we incubate the serum sample with a mixture of biotinylated monoclonal TSH antibody and ruthenium-labeled monoclonal TSH antibody to allow immune complexes formed between the two antibodies and TSH. The solid phase captures these complexes via the streptavidin-coated magnetic microparticles. The chemiluminescence produced by ruthenium upon voltage application will be directly proportional to serum TSH [16,18].

A significant biotin excess in the serum saturates the streptavidin binding sites, thereby resulting in reduced binding of the immune complexes to the solid phase and, hence, a falsely low TSH level [6,8].

The Roche’s (TRAb) bioassay is also a biotin-streptavidin competitive binding assay [3], explaining the elevated levels seen in our case series. The Access TSH bioassay does not use biotin-streptavidin interaction as compared to the Access fT4 and fT3 bioassays, which are competitive binding immune-enzymatic assays, using a monoclonal anti-T4 antibody coupled to biotin and a biotinylated T3 analog, respectively, in their assay design [10,18]. The interference disappears for an immunoassay using no biotin such as the Advia Centaur® (Siemens Healthineers), showing the patient's actual thyroid functions [16].

Older studies had revealed a decrease in the thyroglobulin level in patients taking different doses of biotin because the thyroglobulin bioassay is a sandwich biotinylation-based assay [10,15]. The thyroglobulin level had decreased in the fourth case, but we could not find any explanation for its increase in the third case after biotin discontinuation.

After biotin discontinuation, interference may disappear within eight to 48 hours, but TRAb can take up to seven days to normalize [12,18]. It took 48-72 hours to turn to normal in our patients (Cases 1 and 3).

There is a concern that the results of many other hormonal and non-hormonal immunoassays (troponin, natriuretic peptides, ferritin, tumor markers, therapeutic drug assays, infectious markers, ferritin, human chorionic gonadotropin, steroid and polypeptide hormones, parathyroid hormone, estradiol, testosterone, dehydroepiandrosterone sulfate, etc.) may be misleading because of biotin interference [6,13,19].

## Conclusions

In this case series, the ingestion of the widely used biotin of 20 mg or more for different reasons and different periods led to a potent and clinically relevant thyroid bioassay interference in all the four cases presented here. The clinicians should consider these findings in patients taking biotin supplements of any dose before ordering a thyroid function assay and when interpreting the results. The biotin should be discontinued for at least 48-72 hours before thyroid assaying by any commonly used biotin-streptavidin immunoassay.
